# Supplementary material for: Impact of COVID-19 on Fetal Outcomes in Pregnant Women: A Systematic Review and Meta-Analysis
Source: J Pers Med. 2023 Aug 30;13(9):1337. doi: 10.3390/jpm13091337 (PMC10533032; doi:10.3390/jpm13091337)
Supplement: Supplementary file 1 [file jpm-13-01337-s001.zip › jpm-2571117-supplementary.pptx]

## Slide 1
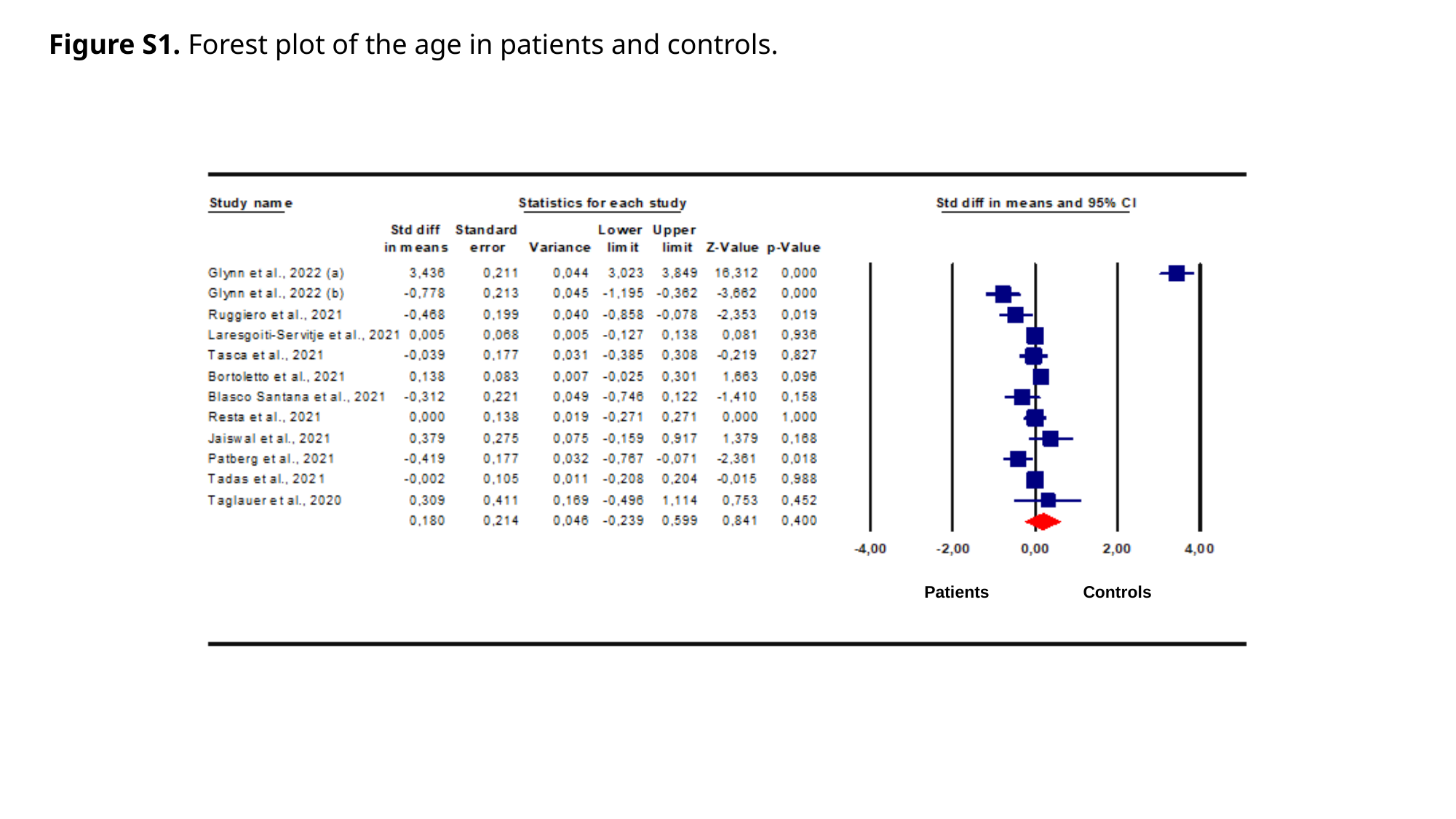

Figure S1. Forest plot of the age in patients and controls.
Patients
Controls

## Slide 2
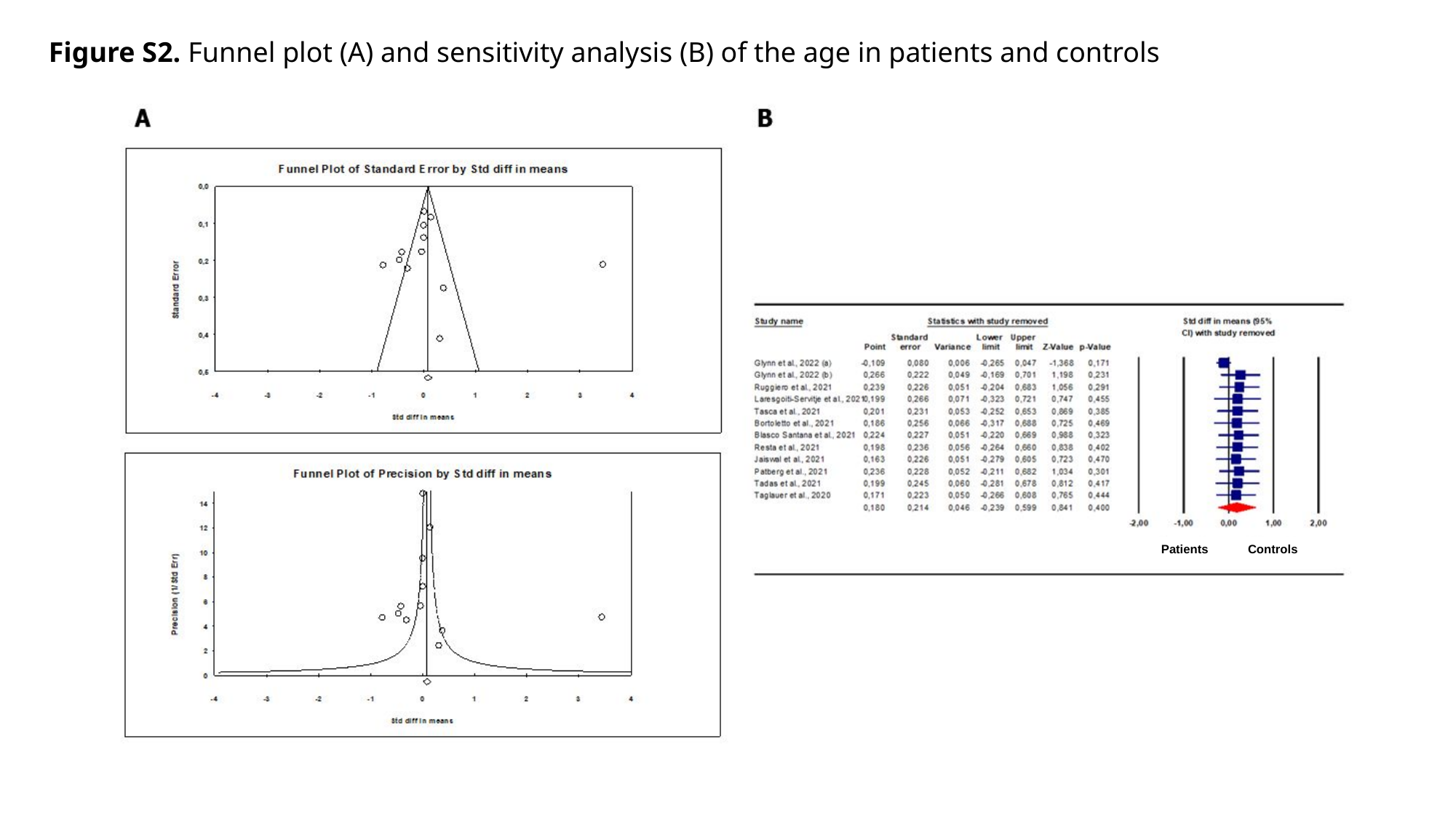

Figure S2. Funnel plot (A) and sensitivity analysis (B) of the age in patients and controls
Patients
Controls

## Slide 3
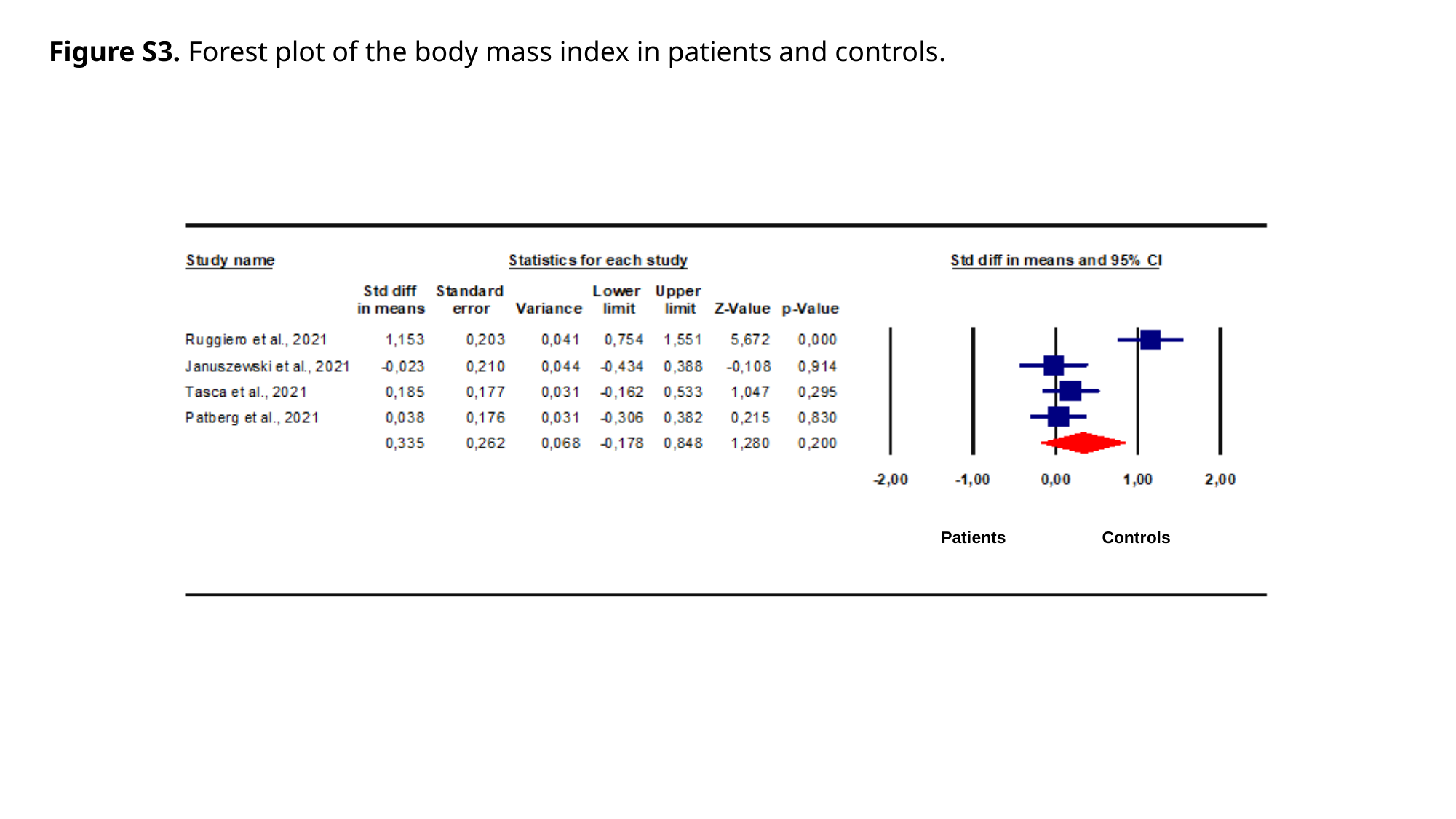

Figure S3. Forest plot of the body mass index in patients and controls.
Patients
Controls

## Slide 4
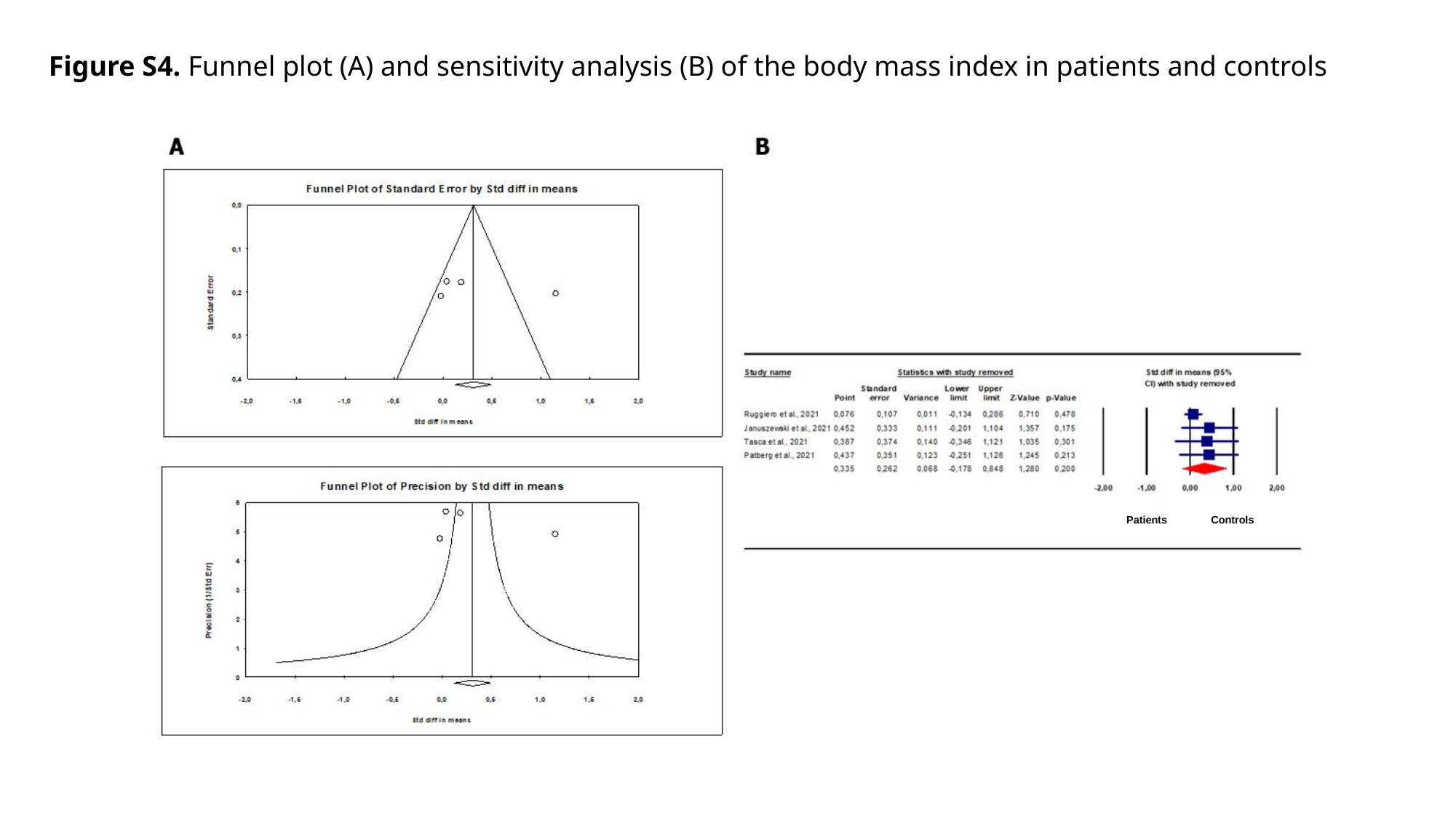

Figure S4. Funnel plot (A) and sensitivity analysis (B) of the body mass index in patients and controls
Patients
Controls

## Slide 5
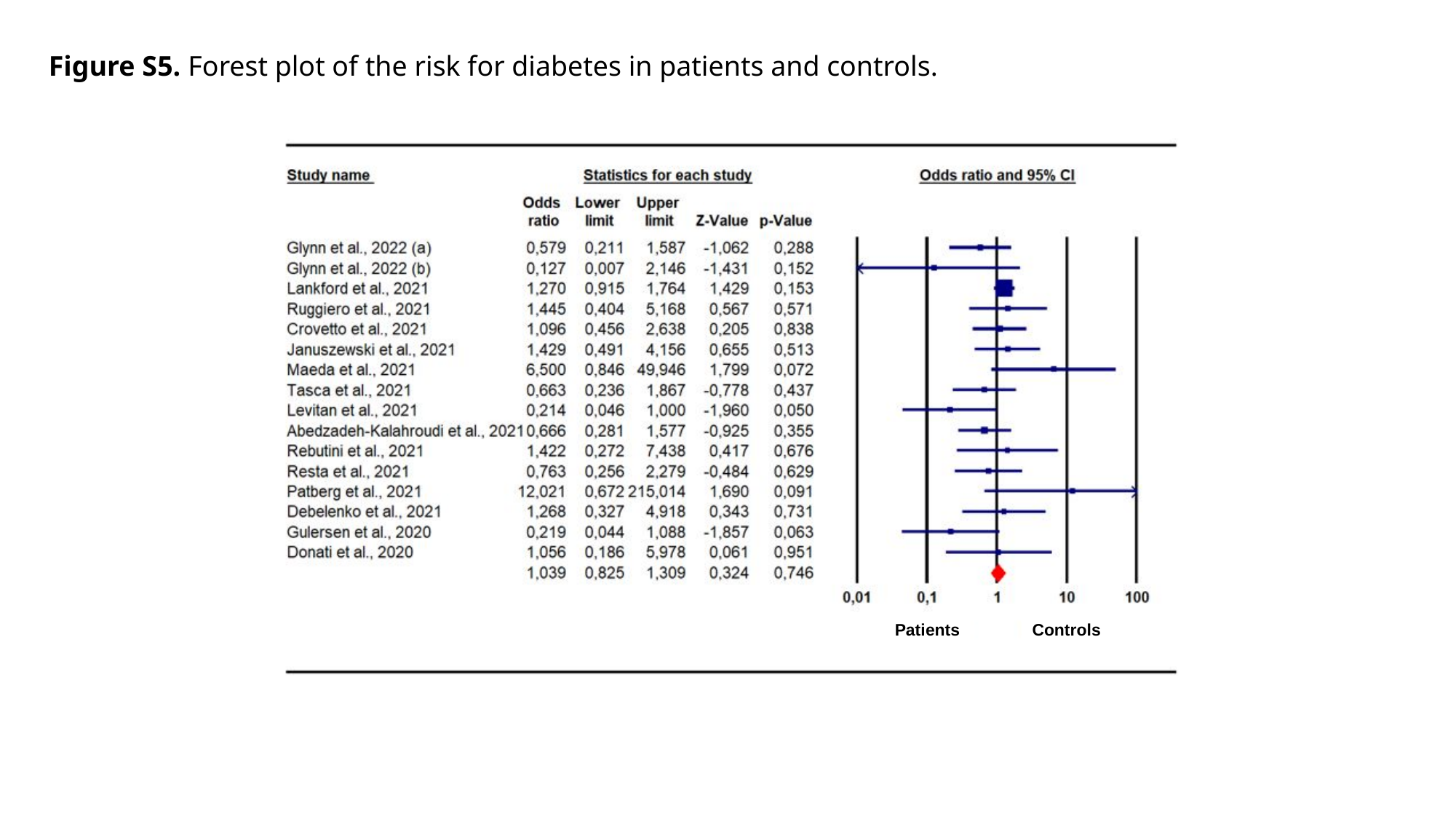

Figure S5. Forest plot of the risk for diabetes in patients and controls.
Patients
Controls

## Slide 6
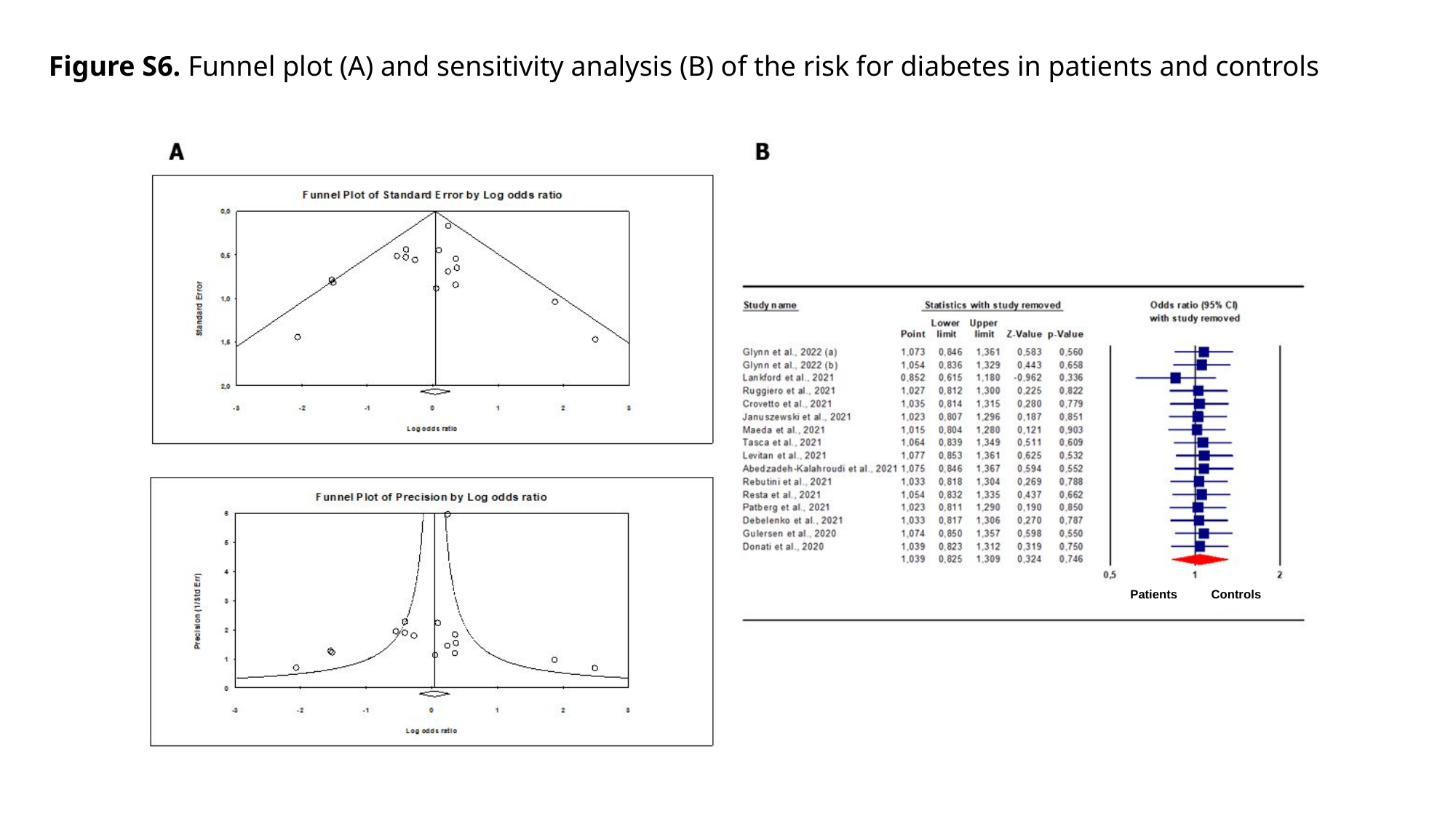

Figure S6. Funnel plot (A) and sensitivity analysis (B) of the risk for diabetes in patients and controls
Patients
Controls

## Slide 7
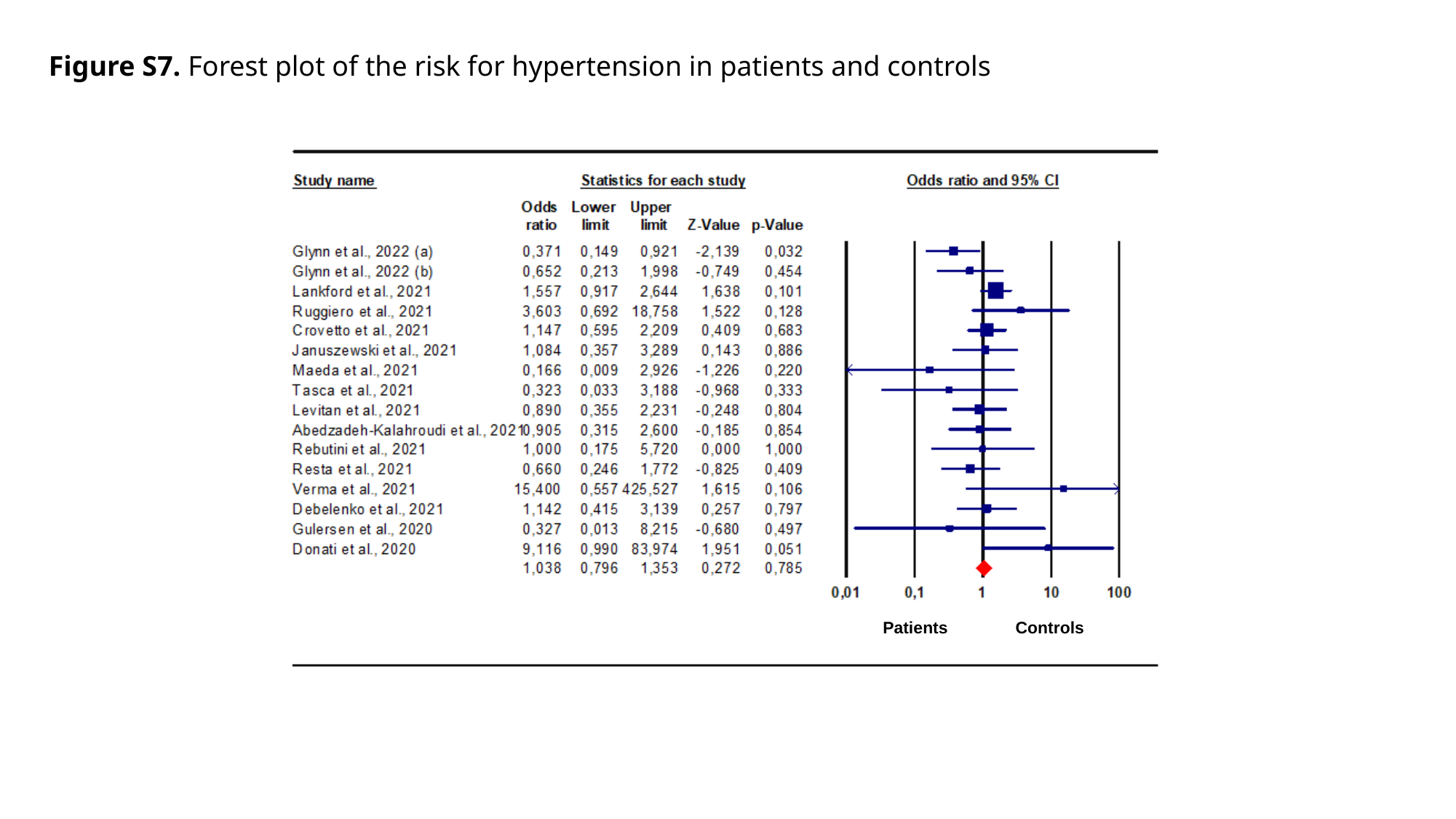

Figure S7. Forest plot of the risk for hypertension in patients and controls
Patients
Controls

## Slide 8
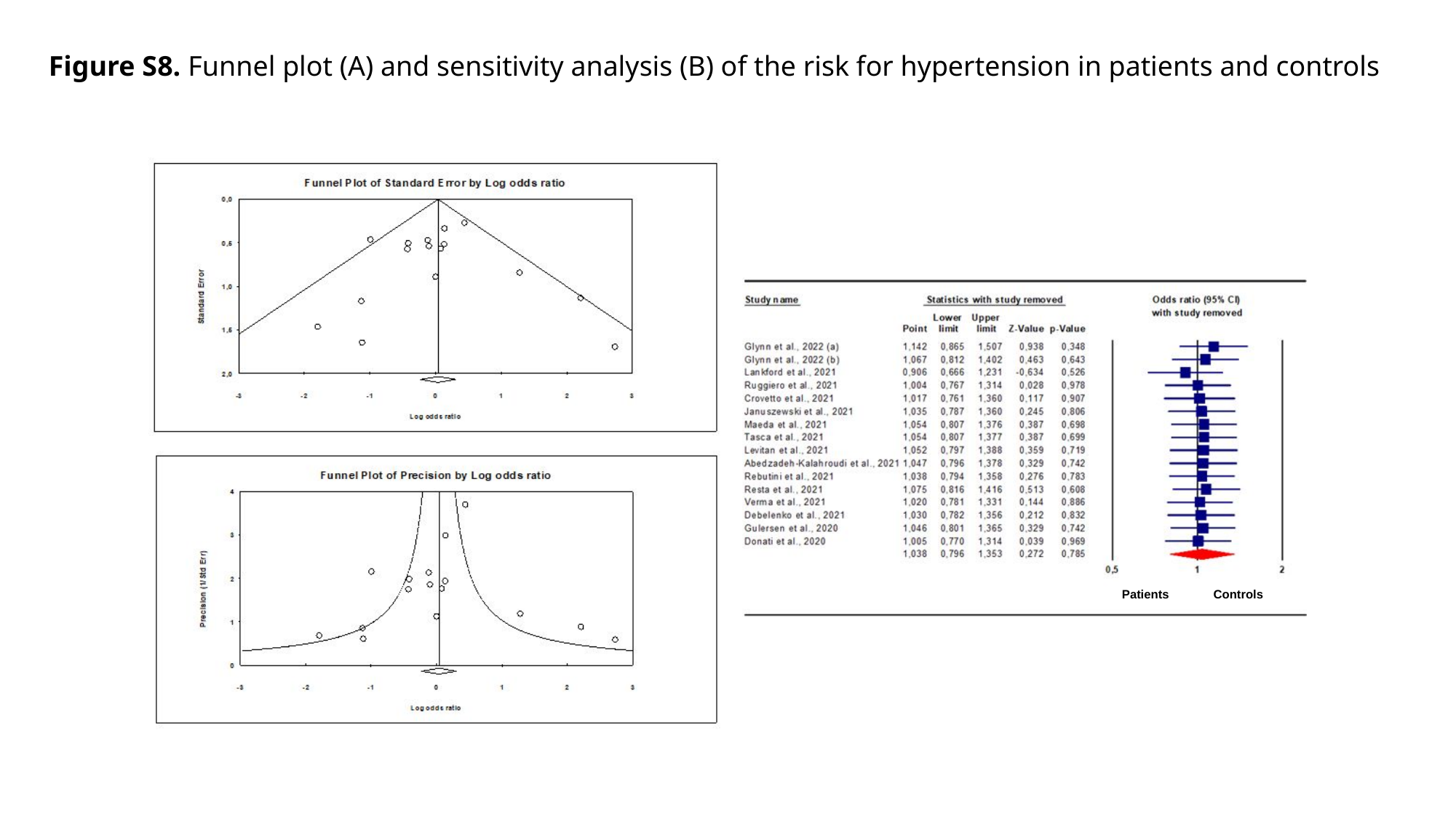

Figure S8. Funnel plot (A) and sensitivity analysis (B) of the risk for hypertension in patients and controls
Patients
Controls

## Slide 9
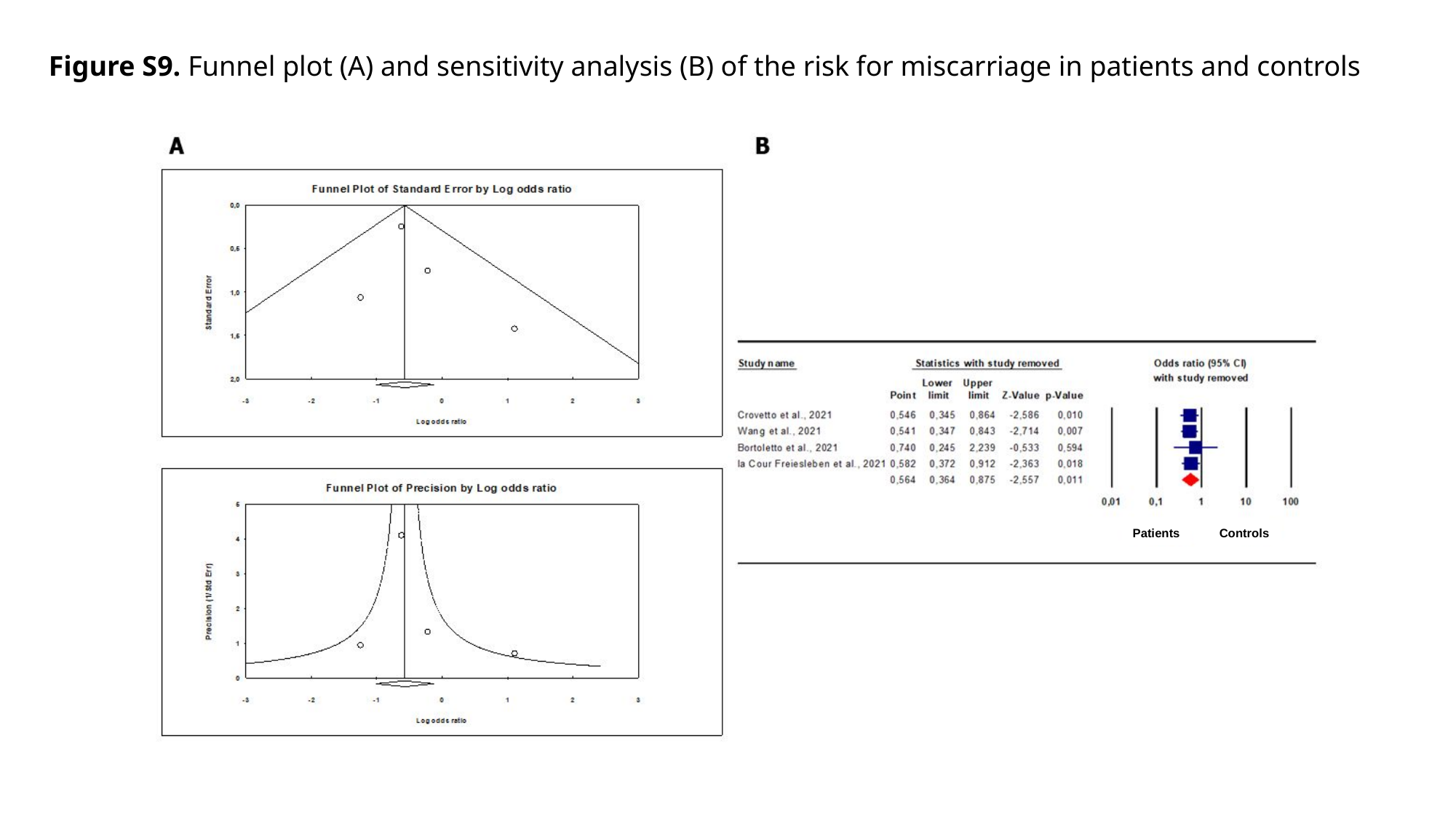

Figure S9. Funnel plot (A) and sensitivity analysis (B) of the risk for miscarriage in patients and controls
Patients
Controls

## Slide 10
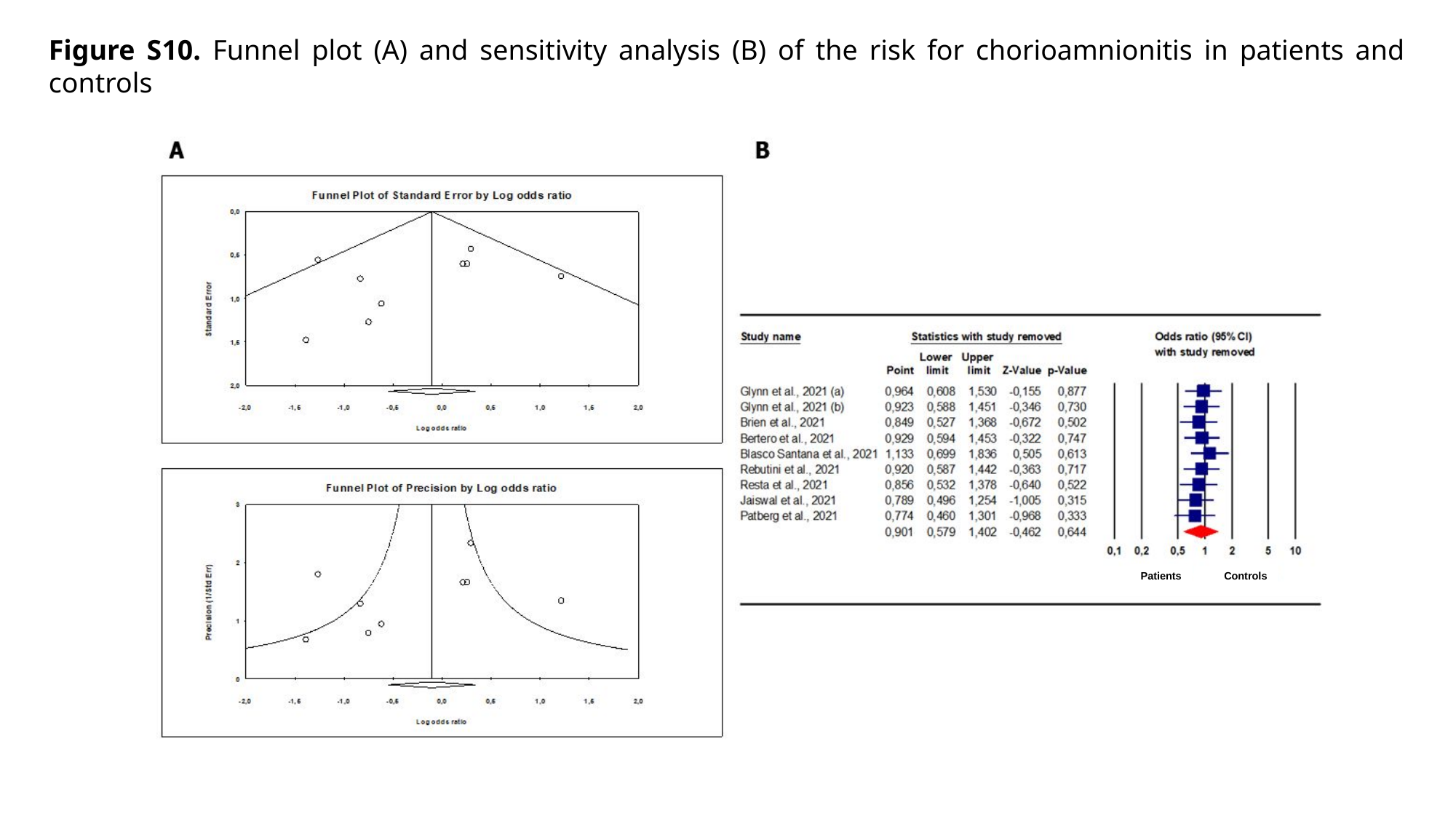

Figure S10. Funnel plot (A) and sensitivity analysis (B) of the risk for chorioamnionitis in patients and controls
Patients
Controls

## Slide 11
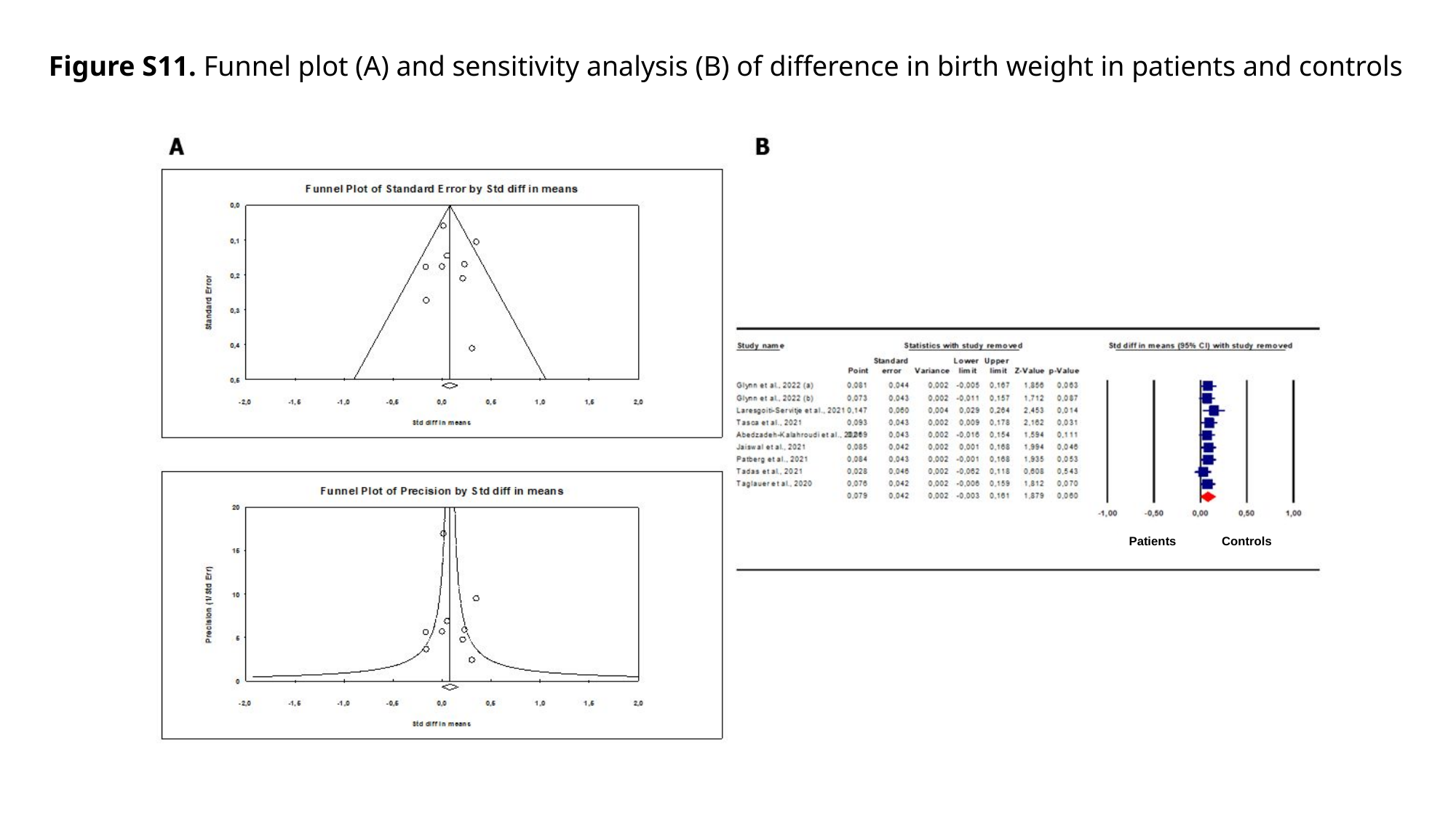

Figure S11. Funnel plot (A) and sensitivity analysis (B) of difference in birth weight in patients and controls
Patients
Controls

## Slide 12
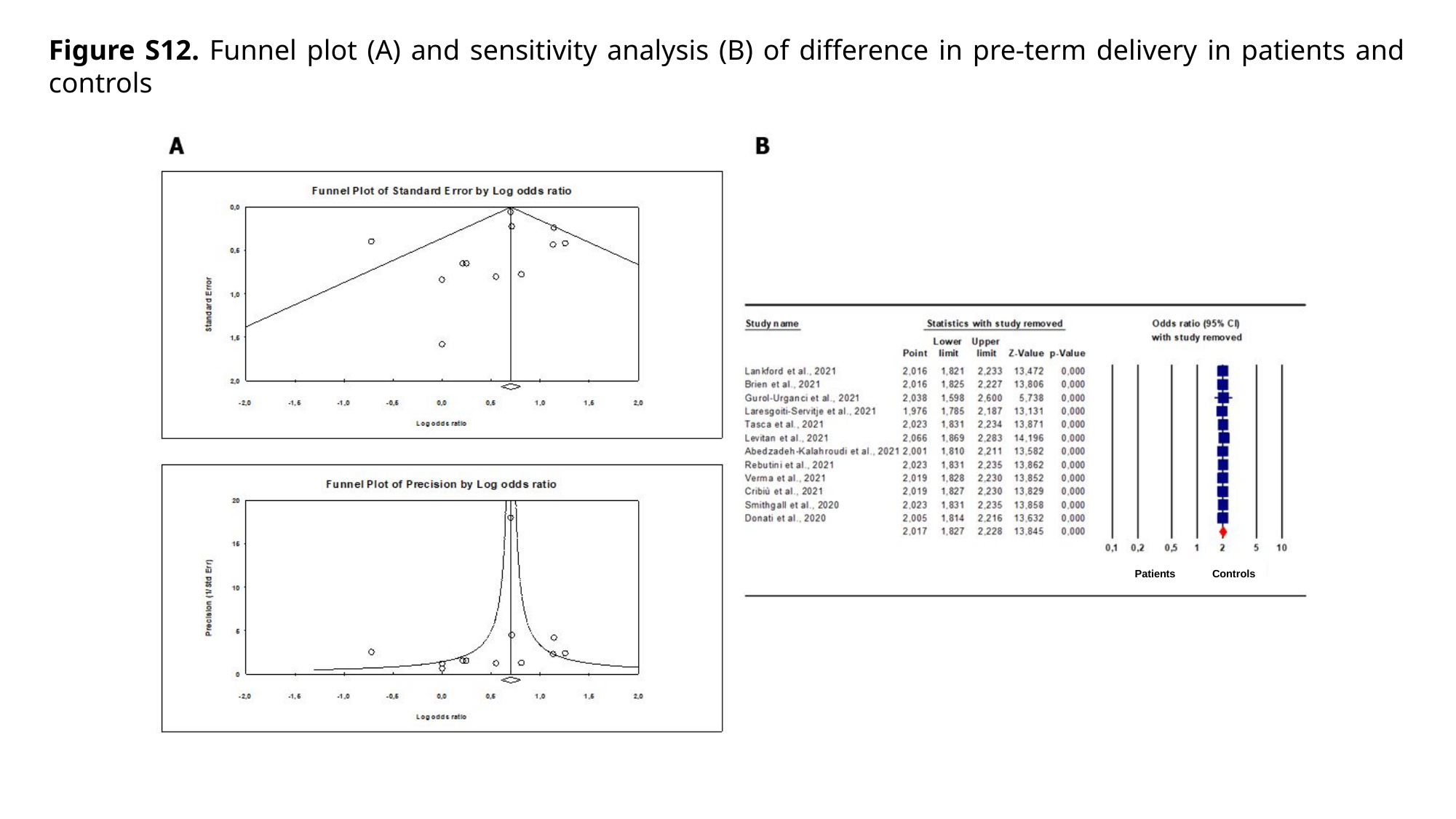

Figure S12. Funnel plot (A) and sensitivity analysis (B) of difference in pre-term delivery in patients and controls
Patients
Controls

## Slide 13
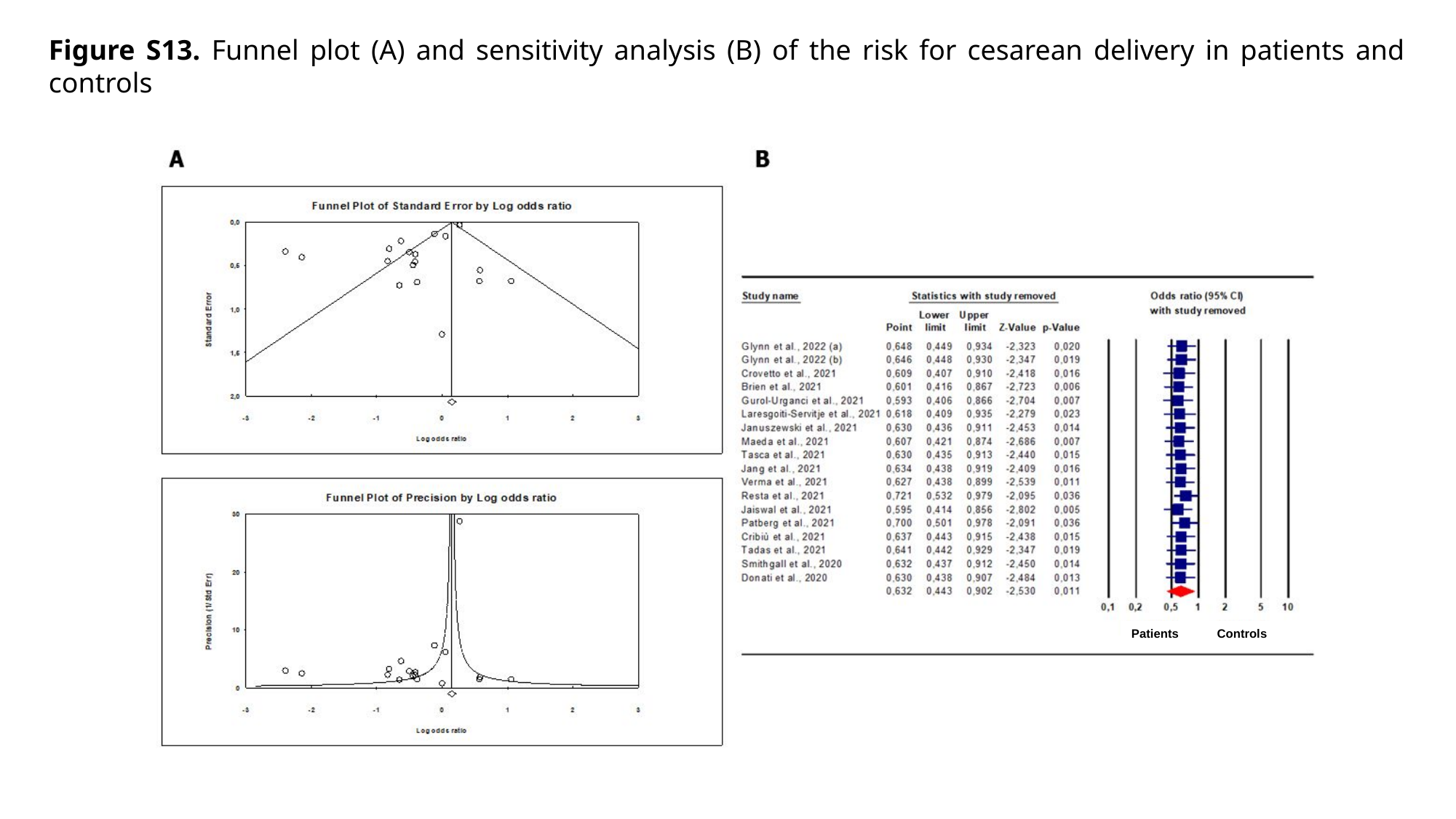

Figure S13. Funnel plot (A) and sensitivity analysis (B) of the risk for cesarean delivery in patients and controls
Patients
Controls

## Slide 14
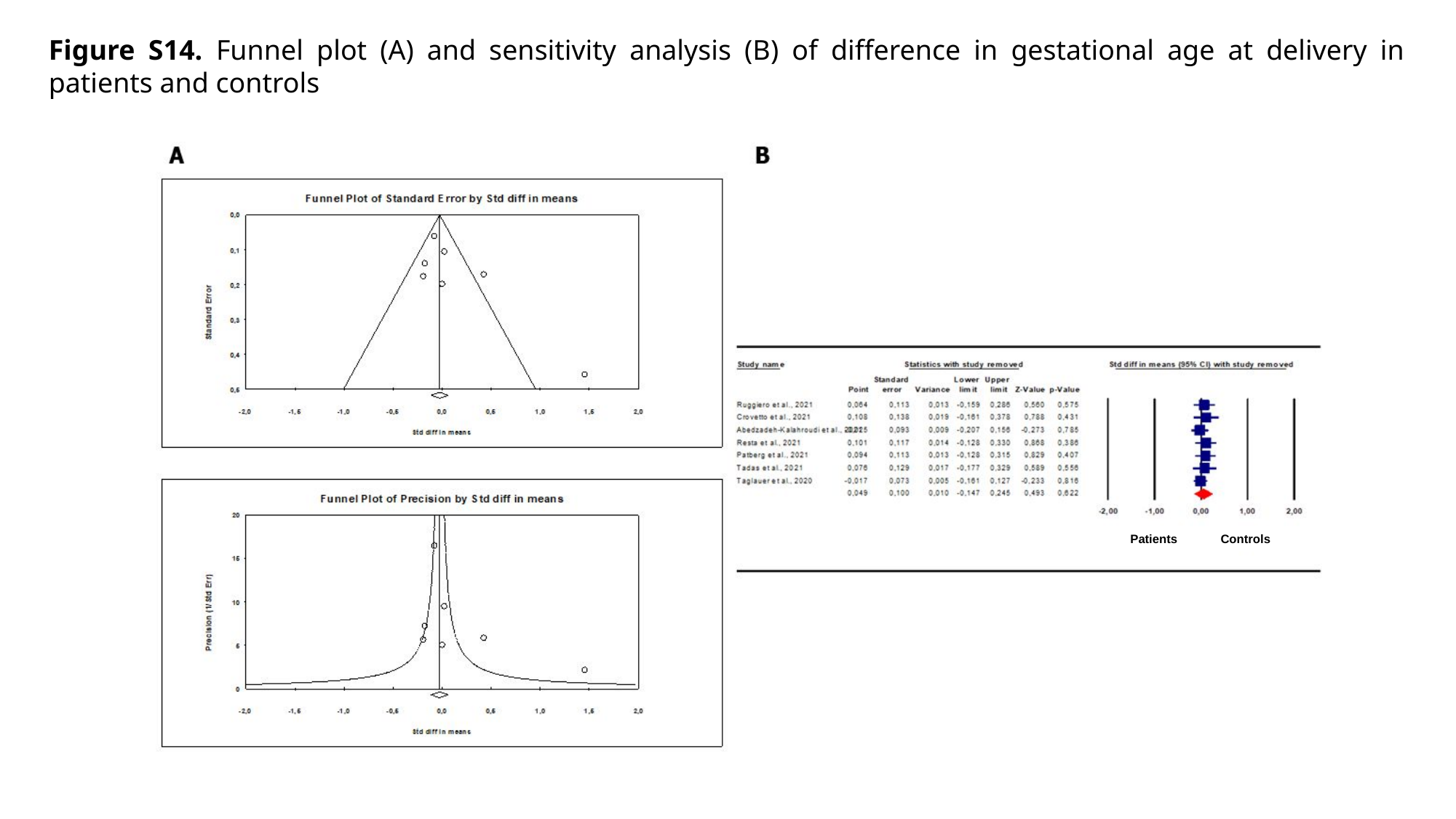

Figure S14. Funnel plot (A) and sensitivity analysis (B) of difference in gestational age at delivery in patients and controls
Patients
Controls

## Slide 15
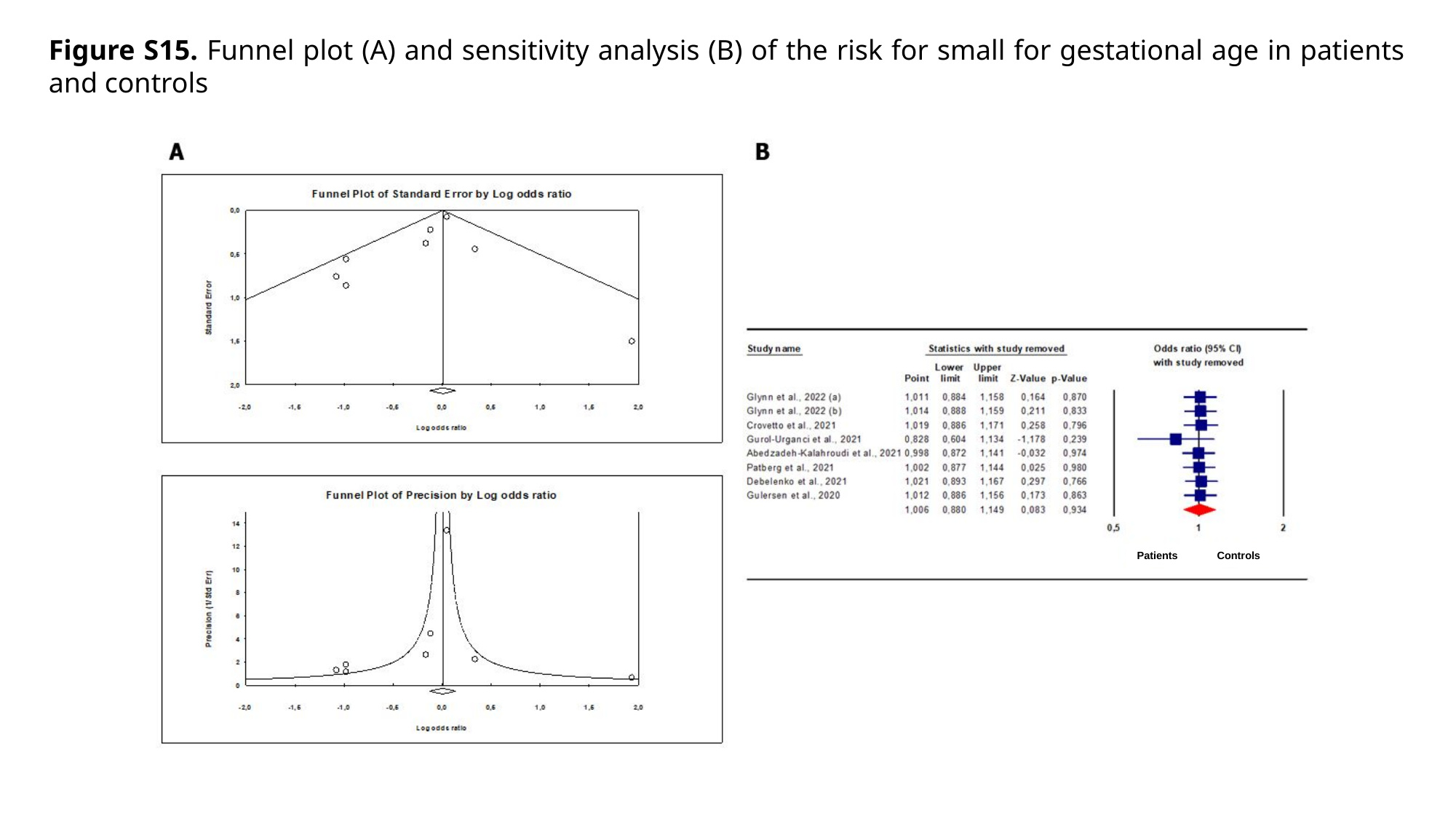

Figure S15. Funnel plot (A) and sensitivity analysis (B) of the risk for small for gestational age in patients and controls
Patients
Controls

## Slide 16
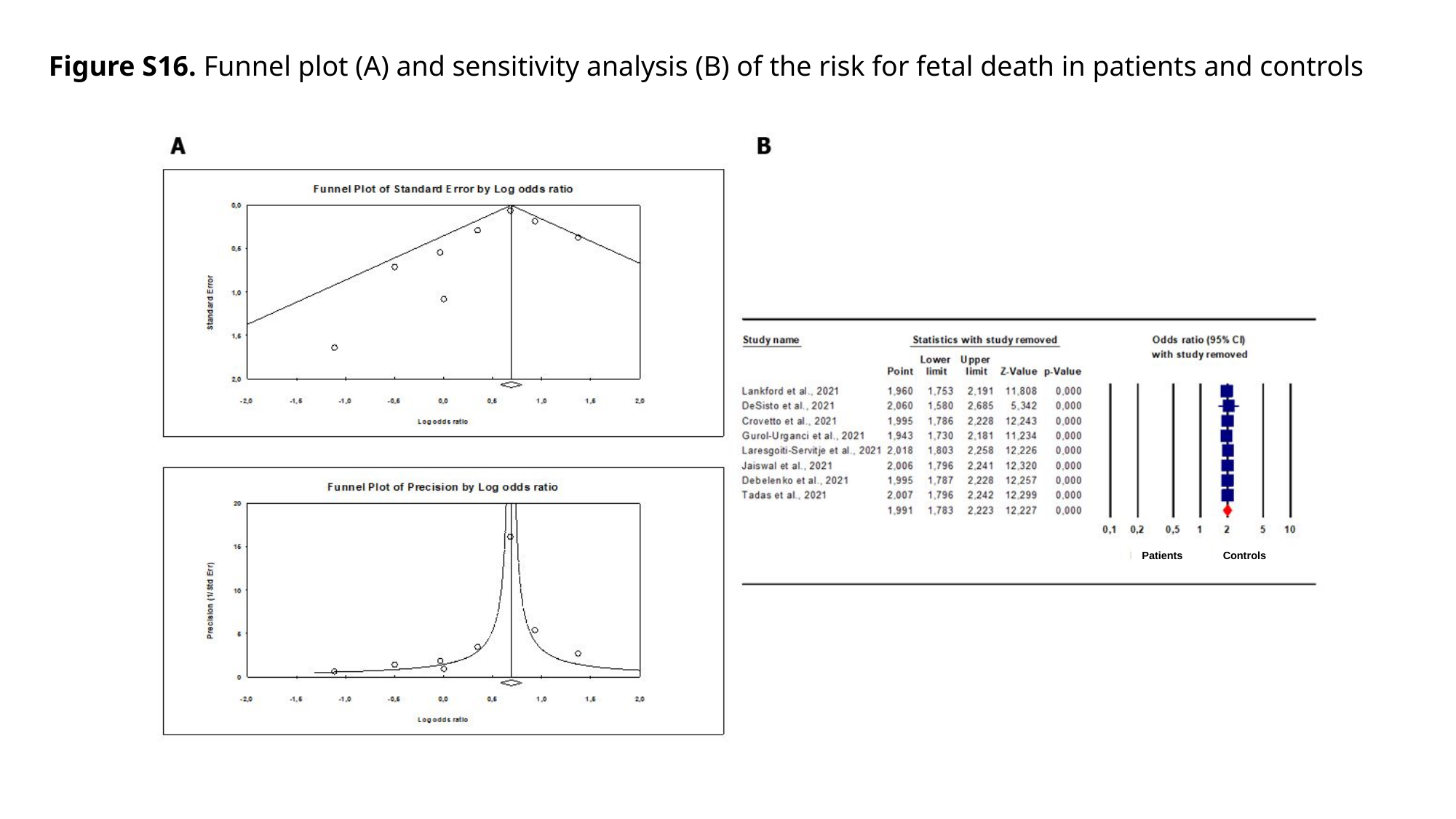

Figure S16. Funnel plot (A) and sensitivity analysis (B) of the risk for fetal death in patients and controls
Patients
Controls
